# Supplementary material for: Shared Treg dysregulation underlies psoriasis vulgaris and atopic dermatitis through the PHF19-PRC2 epigenetic axis
Source: Front Immunol. 2026 Aug 20;17:1932788. doi: 10.3389/fimmu.2026.1932788 (PMC13538097; doi:10.3389/fimmu.2026.1932788)
Supplement: Supplementary file 3 [file DataSheet3.zip › Additional files/Additional file 8/48.CTSS.leaveoneout.pdf]

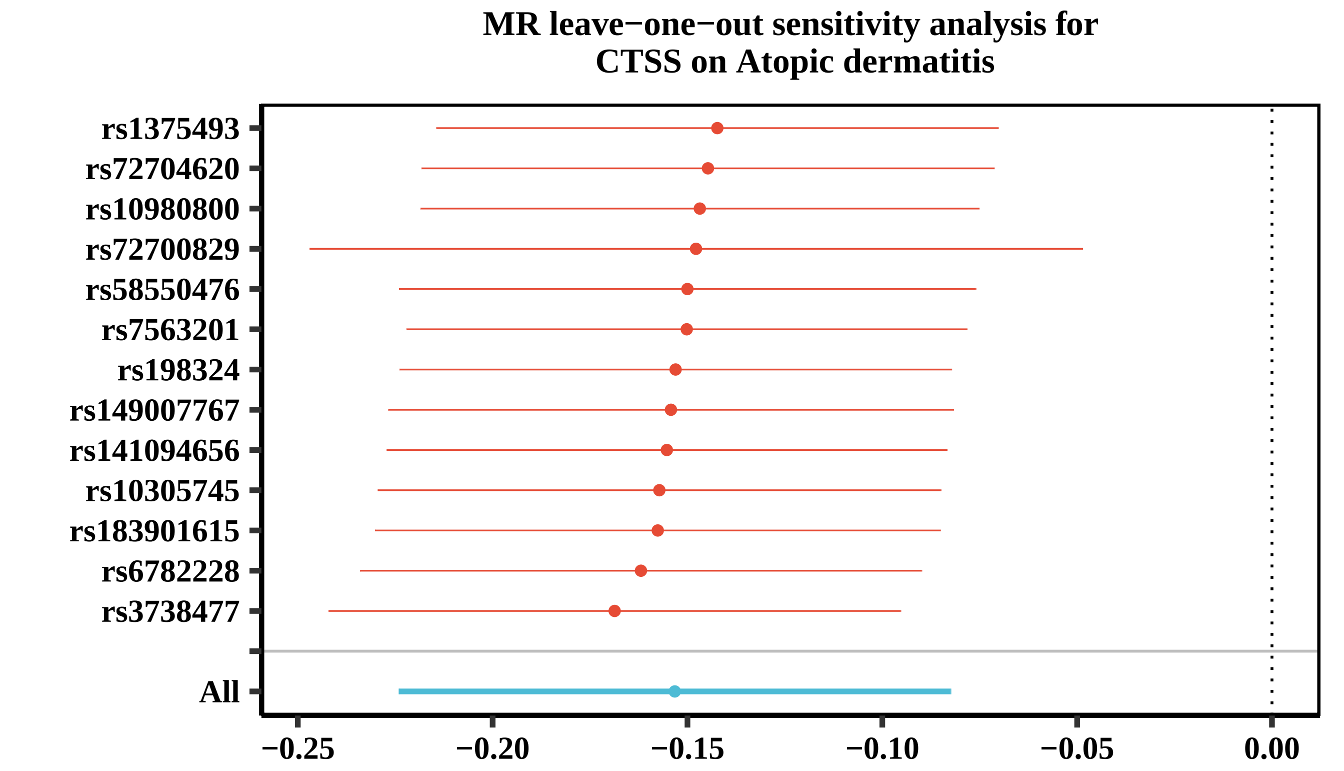
MR leave−one−out sensitivity analysis for
CTSS on Atopic dermatitis
rs1375493
rs72704620
rs10980800
rs72700829
rs58550476
rs7563201
rs198324
rs149007767
rs141094656
rs10305745
rs183901615
rs6782228
rs3738477
All
−0.25
−0.20
−0.15
−0.10
−0.05
0.00
